# Giacomini’s vein – a report on the invaluable importance of an anomalous short saphenous vein

**DOI:** 10.1590/1677-5449.202400242

**Published:** 2024-08-30

**Authors:** Abdalla Ahmed Eldaw Elamin, Naveen Kumar, Vijay Paul Samuel, Kumar Megur Ramakrishna Bhat

**Affiliations:** 1 RAK Medical & Health Sciences University – RAKMHSU, RAK College of Medical Sciences – RAKCOMS, Ras Al Khaimah, United Arab Emirates.; 2 Manipal Academy of Higher Education, Kasturba Medical Collage, Manipal, Karnataka, India.

**Keywords:** Giacomini’s vein, SSV, varicose vein, sural artery flap, veia de Giacomini, veia safena parva, veia varicosa, retalho da artéria sural

## Abstract

Variations in the drainage (termination) and course of the lower limb veins are not uncommon. When dissecting the left lower limb of the adult male cadaver in the vascular case described herein, a unique kind of unilateral short saphenous vein (SSV) termination was observed. It was found that the SSV had normal origin and course in the dorsum of the foot and the back of the leg, respectively. Most often the SSV terminates in the popliteal vein at the popliteal fossa. In this case, it extended upward into the back of the thigh, passing behind the sciatic nerve and then deep to it and the biceps femoris, and finally ended in the veins of the thigh. The SSV did not penetrate any structures along its course to the end, so this unusual vein appears unlikely to be associated with SSV varicose veins. For general, plastic, cardiothoracic, and vascular surgeons, our case would be of significant value.

## Resumo

Variações na drenagem (terminação) e curso das veias dos membros inferiores não são incomuns. No caso vascular relatado, ao dissecar o membro inferior esquerdo de um cadáver adulto do sexo masculino, foi observado um tipo único de veia safena parva (VSP) unilateral. Verificou-se que a VSP tinha origem e curso normais no dorso do pé e na traseira da perna, respectivamente. É mais comum a VSP terminar na veia poplítea, localizada na fossa poplítea. No caso relatado, ela se estendia para cima até a parte posterior da coxa, passando por trás e profundamente no nervo ciático e no músculo isquiotibial, finalizando nas veias da coxa. A VSP não penetra nenhuma estrutura no seu curso; portanto, não é provável que esta veia incomum esteja associada a veias varicosas da VSP. Para cirurgiões gerais, plásticos, cardiotorácicos e vasculares, nosso caso é de extrema importância.

## INTRODUCTION

Developmentally, the short saphenous vein (SSV) is the postaxial vein of the lower limb bud. Its course begins from the lateral marginal vein of the foot and it ascends behind the lateral malleolus and lateral to the calcaneal tendon, accompanying the sural nerve. It generally drains to the popliteal vein just above the level of the knee joint.^1^

Giacomini’s vein (GV) was first described by Carlo Giacomini after observing an extension of the SSV into the thigh. He described as many as eight varieties of short saphenous venous drainage patterns, as reported by Bush and Hammond^2^ (Table 1). Later, de Oliveira et al.^3^ revised the classification, introducing types and sub types (Table 2).

**Table 1:** Giacomini classification of anomalous SSV patterns with reference to the GV, according to Bush and Hammond.

**Type**	**Pattern of thigh extension of SSV, Giacomini classification**
**Type 1**	Presence of a communication channel between GSV and SSV before the SSV drains into the PV
**Type 2**	SSV draining into PV and also extending upwards along the sciatic nerve and terminating in subcutaneous tissue of posterior thigh
**Type 3**	SSV branching as a prominent vein to communicate with the perforators of thigh after draining into PV
**Type 4**	Many short saphenous venous channels draining into perforators
**Type 5**	Whole of SSV draining into GSV through some anastomotic channel
**Type 6**	Few branches terminating into short head of biceps femoris and into GSV through anastomotic vein
**Type 7**	Whole of SSV draining into short head of biceps muscle
**Type 8**	SSV with normal drainage into PV, no thigh extension

SSV: short saphenous vein; GV: Giacomini’s vein; GSV: great saphenous vein; PV: popliteal vein.

**Table 2:** de Oliveira classification^3^ of short saphenous vein based on variant drainage patterns.

Type	SSV drainage pattern	Sub type & drainage pattern
Type 1	Drains into PV	a. Directly to PV
		b. Bifurcates and one division drains into PV and another into GSV
Type 2	Femoral vein/veins of posterior compartment of thigh/into GSV	a. To deep veins of the thigh
		b. Divides and drains into deep veins of the thigh and to GSV
		c. To GSV
Type 3	Into veins of the leg (without reaching PV)	a. Communicates with GSV (at leg region)
		b. Veins of gastrocnemius

SSV: short saphenous vein; PV: popliteal vein; GSV: great saphenous vein.

Recurrent varicosity of the SSV is common following surgery. Ligating the SSV at the saphenopopliteal junction is the common practice when treating a varicose SSV. However, in the presence of Giacomini’s vein, extension of the SSV into the thigh may interfere with this procedure. Venous congestion, which is generally caused by sural arterial flap reconstruction, can be effectively reduced by phlebotomy treatments performed on an intermittent basis in the SSV.^4^

A detailed examination of the presence of variations in the SSV and its termination is necessary since duplex studies of the vein have shown that SSV thigh extensions and their flow pattern can have clinical significance. To ensure a safe and effective intervention, it is imperative to possess adequate knowledge regarding the anatomy, communications with adjacent veins, and pattern of termination of the SSV.

This case report is based on examination of a formalin fixed cadaver, donated for medical education purposes. This study is in compliance with the Helsinki Declaration and with local ethical guidelines. We, the authors, certify that we have obtained all appropriate consent forms and ethics committee clearance for the use of cadavers in this study. No patient data were used in this study

## CASE REPORT

**Figure 1:**
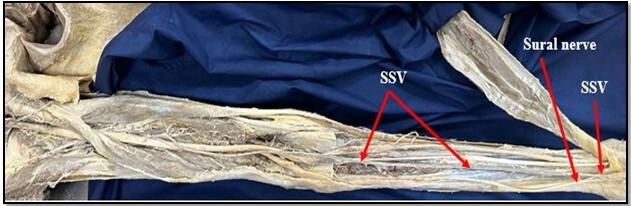
The normal course of the short saphenous vein (SSV) at the back of the leg.

**Figure 2:**
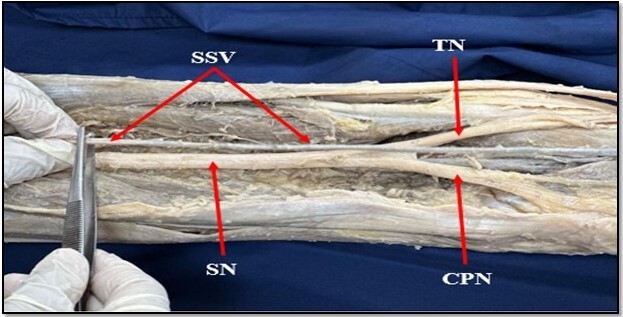
Course of the short saphenous vein (SSV) in the popliteal fossa without communicating with or draining into the popliteal vein and continuing onwards as the Giacomini vein. SN: sciatic nerve; CPN: common peroneal (fibular) nerve; TN: tibial nerve.

**Figure 3:**
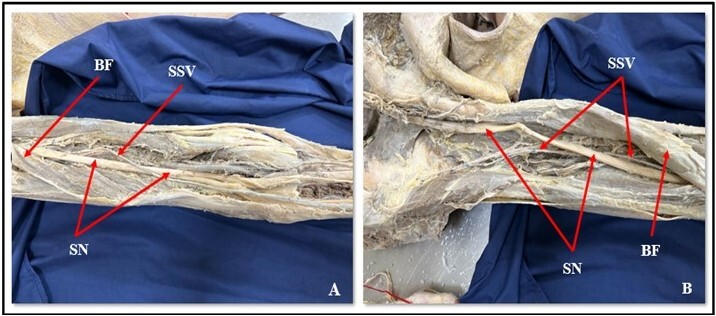
(A) Further course of Giacomini vein in the middle of the thigh and (B) its course in relation to the structures of the back of the thigh. SN: sciatic nerve; BF: biceps femoris; SSV: short saphenous vein.

**Figure 4:**
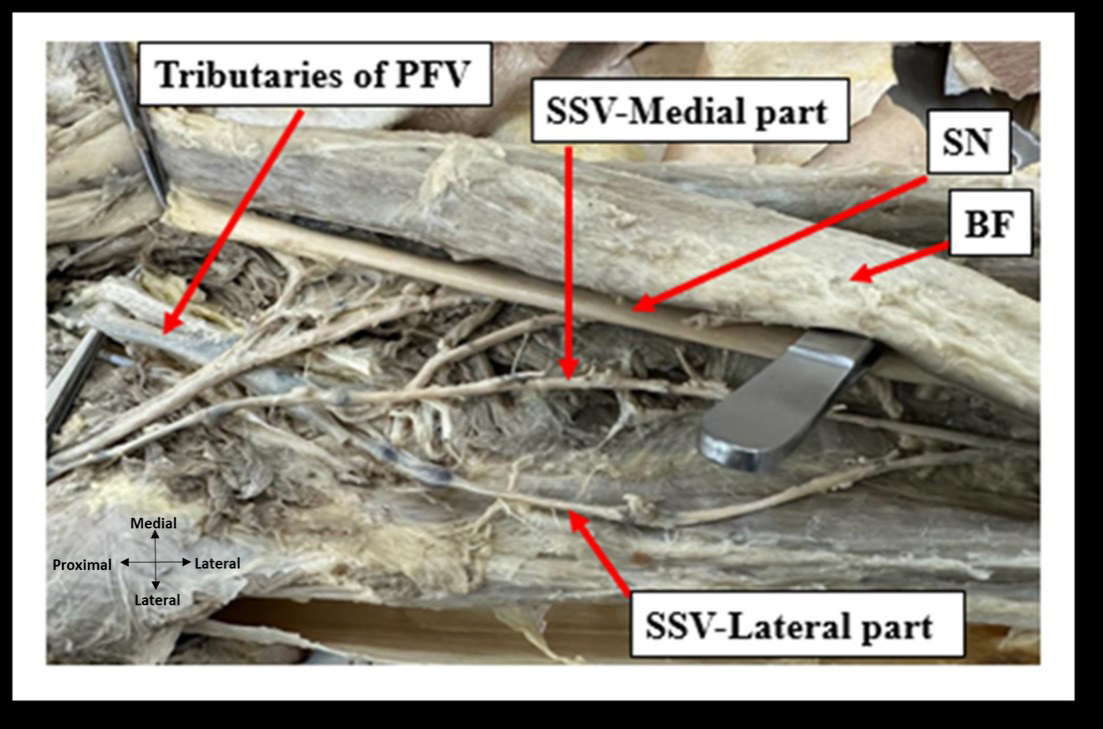
Termination of the Giacomini variant of the short saphenous vein (SSV) into the tributaries of the profunda femoris vein (PFV) and the veins of the thigh. SN: sciatic nerve; BF: biceps femoris.

The venous variation presented herein was observed in an adult male cadaver aged around 60 years, during routine dissection of the left lower limb. We found an uncommon form of termination of the left SSV. The SSV had its normal commencement from the lateral end of the dorsal venous arch in the dorsum of the foot and the normal course along the posterior aspect of the leg accompanied by the sural nerve (Figure 1). In its further course, instead of draining to the popliteal vein in the popliteal fossa, it crossed the fossa superficially to continue in the lower half of the posterior part of the thigh as the vein of Giacomini, positioned behind the sciatic nerve **(**Figure 2). On approaching the middle of the thigh, the SSV curved medially and traversed deep (anterior) to the sciatic nerve and the long head of the biceps femoris (Figure 3). In the upper posterior part of the thigh, it divided into lateral and medial divisions. The former terminated in the tributaries of the profunda femoris vein (PFV) while the latter ended in the veins of the back of the thigh (Figure 4). Along its course, it was observed that it maintained communication with the long saphenous vein, but no communication with the popliteal vein was seen.

## DISCUSSION

Insufficiency of the short saphenous vein (SSV) was formerly regarded as less important. However, increased ultrasound scanning studies of varicose vein disease have revealed a link between downward reflux of varicose disease and the SSV.^5^ Therefore, any anomalous morphology of the SSV has great clinical importance as it can contribute to recurrent varicose veins if the proper investigation is neglected.^6^

During vasculogenesis, the cervical and lumbar intersegmental vessels route towards their respective extremities and begin to form the anastomosing channels. By the commencement of the single axial artery, venous blood flows to the heart through the cardinal venous system via preaxial and postaxial veins which eventually form great and short saphenous veins respectively in developing lower limbs. Alterations in their course result in variations.^7^ The SSV and Giacomini veins (GV) share similar development from the primitive vessel, accompanying the sural nerve, and also course in the same interfascial partition.^8^

The scientific literature has a sufficient number of reports on varied SSV drainage patterns. Nevertheless, a precise categorization of drainage or communication patterns remains to be determined. Several researchers have suggested distinct methods for categorization.

The prevalence of GV is highly variable and is reported to be 2% according to studies detecting GV by duplex scanning. ^9^ Hence, it is highly recommended to perform a duplex ultrasound scan before undertaking any lower extremity surgery in patients with chronic venous illness to rule out the possibility of GV. The occurrence of detection of SSV thigh extension is increasing with duplex scanning. A recent study revealed that the majority of variant SSV terminations were to deep veins of the mid thigh and few were to the superior gluteal vein.^10^

The existence of a Giacomini vein poses several clinical problems arising from its incompetence. It may or may not be associated with varicosity of saphenous veins. Short saphenous vein reflux makes a significant contribution to the various different etiological causes of chronic venous insufficiency (CVI), with an incidence rate as high as 15%.^11^ Unfortunately, SSV reflux and incompetence is generally ignored in the management of CVI and often misinterpreted as that of the great saphenous vein. This neglect may eventually lead to deep vein thrombosis or varicose vein recurrence.^12^

Vascular surgeons should be well aware of this fact and be cautious, particularly during preoperative ultrasound scanning procedures.^13^ A Giacomini vein could be chosen for autologous grafting when the great saphenous vein is inaccessible and also serves as an alternate venous conduit for venous grafting. The Giacomini vein is considered the most suitable alternative choice in arterial bypass surgery for arterial reconstruction when the great saphenous vein is unsuitable to be harvested.^14^ Hence, it is imperative to be familiar with any varied drainage pattern of the SSV before choosing it for the graft or for any other surgical procedures. Preoperative duplex scanning to ascertain possible persistence of the GV or anomalous SSV is essential in diagnosis of chronic venous insufficiency and deep vein thrombosis and in the proper management of varicosity.

## CONCLUSION

Due to the intricacy and frequent anatomic variations of veins, venous ultrasonography has become one of the most difficult procedures for sonographers to perform. Existence of a Giacomini vein poses several clinical problems arising from its incompetence. It may or may not be associated with varicosity of the saphenous veins. Nevertheless, presence of a GV would be of significant value for general, cardiothoracic, and plastic surgeons.
